# Application of Highly Sensitive Immunosensor Based on Optical Waveguide Light-Mode Spectroscopy (OWLS) Technique for the Detection of the Herbicide Active Ingredient Glyphosate

**DOI:** 10.3390/bios13080771

**Published:** 2023-07-29

**Authors:** Krisztina Majer-Baranyi, Fanni Szendrei, Nóra Adányi, András Székács

**Affiliations:** 1 Food Science Research Group, Institute of Food Science and Technology, Hungarian University of Agriculture and Life Sciences, Villányi út 29-43, H-1118 Budapest, Hungary; majerne.baranyi.krisztina@uni-mate.hu; 2 Institute of Isotopes Co., Ltd., Konkoly-Thege Miklós út 29-33, H-1121 Budapest, Hungary; 3 Agro-Environmental Research Centre, Institute of Environmental Sciences, Hungarian University of Agriculture and Life Sciences, Herman Ottó út 15, H-1022 Budapest, Hungary; szekacs.andras@uni-mate.hu

**Keywords:** glyphosate, immunosensor, label-free detection, OWLS

## Abstract

The herbicide active ingredient glyphosate is the most widely applied herbicidal substance worldwide. Currently it is the market-leading pesticide, and its use is projected to further grow 4.5-fold between 2022 and 2029. Today, glyphosate use exceeds one megaton per year worldwide, which represents a serious environmental burden. A factor in the overall boost in the global use of glyphosate has been the spread of glyphosate-tolerant genetically modified (GM) crops that allow post-emergence applications of the herbicide on these transgenic crops. In turn, cultivation of glyphosate-tolerant GM crops represented 56% of the glyphosate use in 2019. Due to its extremely high application rate, xenobiotic behaviour and a water solubility (11.6 mg/mL at 25 °C) unusually high among pesticide active ingredients, glyphosate has become a ubiquitous water pollutant and a primary drinking water contaminant worldwide, presenting a threat to water quality. The goal of our research was to develop a rapid and sensitive method for detecting this herbicide active ingredient. For this purpose, we applied the novel analytical biosensor technique optical waveguide light-mode spectroscopy (OWLS) to the label-free detection of glyphosate in a competitive immunoassay format using glyphosate-specific polyclonal antibodies. After immobilising the antigen conjugate in the form of a glyphosate conjugated to human serum albumin for indirect measurement, the sensor chip was used in a flow-injection analyser system. For the measurements, an antibody stock solution was diluted to 2.5 µg/mL. During the measurement, standard solutions were mixed with the appropriate concentration of antibodies and incubated for 1 min before injection. The linear detection range and the EC_50_ value of the competitive detection method were between 0.01 and 100 ng/mL and 0.60 ng/mL, respectively. After investigating the indirect method, we tested the cross-reactivity of the antibody with glyphosate and structurally related compounds.

## 1. Introduction

Glyphosate has become the most dominant non-selective broad-spectrum herbicide worldwide since its introduction in 1974. Since then, the amount used each year has been continuously increasing, mainly since the spread of glyphosate-resistant genetically modified (GM) crops [1]. According to a survey launched in 2019, the total sales of glyphosate were estimated at 46,527 tonnes in 2017 across the EU, even though glyphosate-resistant genetically modified crops are not cultivated in the EU, which represented 33% of total herbicide sales [2]. It is used for weed control on industrial and on agricultural land in pre-emergent and post-cropping of fields, as a ‘harvest aid’ for desiccation on traditional grain crops and also in urban areas such as on roadsides and beside railway tracks, along streets and parks. In regions where the cultivation of glyphosate-resistant GM crops is authorised, increased application occurs due to the fact that the genetic modification in these crops makes post-emergent applications possible [2]. Glyphosate is taken up rapidly through plant surfaces such as leaves and translocates to growing sites (meristems, young roots, storage organs) where it inhibits the enzyme 5-enolpyruvyl-shikimate3-phosphate synthase (EPSPS) of the shikimate pathway required for the biosynthesis of all three aromatic amino acids (phenylalanine, tyrosine and tryptophan). Blocking the biosynthesis of these essential amino acids results in rapid plant necrosis and death after several days [3]. Glyphosate is usually marketed as an isopropyl aminonium, potassium, ammonium or trimethylsulfonium salt and is formulated with various adjuvants to enhance the uptake and translocation of the active ingredient in plants. These adjuvant compounds are claimed to enhance the cytotoxic properties of glyphosate [4,5]. Glyphosate adsorption is typically strong on soil particles, therefore the compound was considered to have the lowest mobility potential in soil among pesticides. Nonetheless, despite this high sorption tendency on soil particles, several studies demonstrated that glyphosate can be washed into deeper soil layers and it leaches with drainage water, contaminating surface or ground waters. In turn, the exposure of non-target organisms, both plants and animals (especially aquatic organisms), is of great concern in ecotoxicology [6,7].

Glyphosate is quite resistant to degradation. Its half-life could range from a few days to a few months or even years in nature. Its main decomposition pathway in soil and water is through microbial degradation under both aerobic and anaerobic conditions. The main intermediate metabolites of glyphosate metabolism are aminomethylphosphonic acid (AMPA), sarcosine and acetylglyphosate, which are further metabolised through different metabolism pathways [8]. The main metabolite, AMPA, is more mobile in soil than the parent compound, therefore both glyphosate and AMPA leaching to surface and ground water pose a great threat to water quality. In several countries, glyphosate has been detected in surface and ground water not only from agricultural areas but also from natural protected areas. Mörtl et al. [9] reported glyphosate concentrations ranging from 0.54 to 0.76 ng/mL in ground water samples in Hungary. In Argentina, glyphosate levels of surface waters ranged from 0.10 to 0.70 µg/mL were detected by Peruzzo et al. [10]. In Colombia, 39.4% and 55% of all environmental samples were contaminated with glyphosate and AMPA with a maximum concentration of 0.48 and 0.40 µg/mL, respectively, according to the survey of Battaglin et al. [7].

Exposure to glyphosate and AMPA has been shown to induce antibiotic resistance in soil bacteria and may result in shifts in microbial communities in soil, plants, water and the intestinal tracts of animals and humans [11,12]. Toxicity to honeybees, birds, amphibians and fishes has also been documented [4,13,14]. Moreover, carcinogenic, teratogenic, hepatorenal and endocrine disruption effects due to the exposure to glyphosate-based herbicides, even at low concentrations, have also been reported [15,16,17,18]. The International Agency for Research on Cancer (IARC) classified glyphosate as probably carcinogenic to humans, based on ‘limited evidence’ in human experiments and ‘sufficient evidence’ in animal experiments [19]. To eliminate any potential risk to human health, maximum residue limits (MRL) have been set for glyphosate in drinking water (0.1 ng/mL) and in food, depending on the product: 0.05 mg/kg for milk, eggs and honey, 20 mg/kg for barley, oat, sorghum, soybean and sunflower seed; 10 mg/kg for wheat, rye, cotton seed, linseed, mustard seed, rapeseed, lentils, lupins and peas; 2.0 mg/kg for beans; 1.0 mg/kg for corn; and 0.1 mg/kg for most other cereals and grains if not specified [20].

Due to their low molecular weight, high polarity, zwitterionic structure, insolubility in organic solvents, low volatility and thermal lability, glyphosate and its main metabolite AMPA are difficult to separate using liquid chromatography or gas chromatography analysis, as these properties cause problems in extraction, purification and determination [21]. Moreover, the analytical methods developed require derivatisation. Currently preferred techniques for glyphosate determination are based on liquid chromatography coupled to a variety of detectors, such as high-performance liquid chromatography with fluorescence detection (HPLC-FLD), ultraviolet detection (HPLC-UV) [22], liquid chromatography–tandem mass spectrometry (LC-MS/MS) [23] and liquid chromatography using solid-phase extraction coupled to mass spectrometry with electrospray ionisation (LC–SPE–ESI/MS/MS) [24,25]. In addition to these techniques, the determination of glyphosate using ELISA or immunosensor techniques is increasingly coming to the fore by taking advantage of the selectivity of the biological recognition elements [26,27,28].

In recent decades, the use of biosensors in the field of environmental analytics has gained significant attention. Optical immunosensors, known for their sensitivity, have proven to be effective in the detection of small-molecule pollutants such as pesticide residues [29]. One particular technique used in these sensors is optical waveguide light-mode spectroscopy (OWLS), which enables in situ and label-free investigation of surface processes at the molecular level by utilising the evanescent field formed above the sensor [30]. OWLS relies on the precise measurement of the resonance angle of polarised laser light (wavelength: 632.8 nm), which is diffracted by the waveguide grating and coupled into a thin waveguide on the sensor surface. The coupling resonance can be accurately measured based on the optical parameters of the sensor chips and the complex refractive index of the sample medium covering the sensor. The intensity of light coupled by multiple internal reflections within the waveguide layer is measured using photodiodes. By determining the refractive index from the resonance coupling angle, the thickness and coverage (or mass) of the adsorbed or surface-bound material can be determined with high sensitivity. The technique has a limit of detection (LOD) of a few pg/mm^2^. This method is suitable for the developing chemosensors and biosensors, allowing the direct detection of various biomolecules when bioactive substances are attached to the surface of the waveguide. The aim of the present study was to develop a label free, OWLS technique-based detection method for glyphosate determination in a competitive immunoassay format as an alternative approach to the drawbacks exposed in other techniques, such as tedious, time-consuming sample pre-treatments, high-cost end equipment and large amounts of highly pure organic solvents and long analysis times. OWLS enables in situ and label-free investigation of surface processes at a molecular level using an evanescent field. The technique combines the high selectivity characteristic of immunoreactions with high sensitivity determination, which allowed the technique to be used for the determination of many compounds, including the determination of small molecules [31,32,33,34].

## 2. Materials and Methods

### 2.1. Materials

Organic chemicals and solvents, glyphosate standard and structurally similar compounds and salts for buffers were purchased from Sigma-Aldrich Inc. (St. Louis, MO, USA). The purity of standard solutions was ≥98%. Other reagents of analytical grade were purchased from VWR (Debrecen, Hungary). The waveguide sensors (chips) type OW 2400 were purchased from MicroVacuum (Budapest, Hungary). The development of the competitive immunosensor involved the conjugation of the succinylated GLY-analogue to HSA at the Institute of Isotopes Co., Ltd., (Budapest, Hungary) Subsequently, the final product underwent dialysis and lyophilization before being used to sensitise the sensor surface. The ELISA kit for glyphosate detection (EK-102M200506) was obtained from the Institute of Isotopes Co., Ltd. (Budapest, Hungary). The primary polyclonal glyphosate antibody produced in chicken (anti-GLY) was purchased from Agrisera AB (Vannas, Sweden). Goat anti-chicken immunoglobulin conjugated to horseradish peroxidase (HRP) as a secondary antibody was obtained from TS Labor (Budapest, Hungary). Immunoassays were carried out in sterile 96-well Costar HB microplates (Corning Inc., Corning, NY, USA) in a fluorescent assay format.

### 2.2. OWLS Instrument Set-Up

The OWLS measurement was carried out using OWLS 210 instrument controlled by software BioSense 3.7 (MicroVacuum Ltd., Budapest, Hungary). For measurements, an integrated optical waveguide sensors (chips) type OW 2400 with optical grating of 2400 lines per mm in the SiO_2_-TiO_2_ waveguide layer (MicroVacuum Ltd., Budapest, Hungary) was used. The sensor chip was illuminated using a polarised He-Ne laser light (632.8 nm) vertically from below, while the sensor chip was rotated continuously in a narrow (±5°) angle. Provided that the incoupling condition is fulfilled, the light is coupled by the diffraction grating into the waveguide layer where it propagates by the total internal reflection. The incoupled light intensity is monitored at the edges of the waveguide using photodiodes. Incoupling is a resonance phenomenon that occurs at well-defined angles depending on the refractive indices of the covering medium of the waveguide. Knowing the incoupling angles, effective refractive indices can be calculated from which the thickness of the adsorbed molecules deposited on the surface and the surface coverage can be calculated.

All determinations were carried out at room temperature in a flow-injection analyser (FIA) system containing a low-pulse peristaltic pump (Minipuls 3, Gilson Inc., Middleton, WI, USA), maintaining the flow rate of 160 µL/min, and an injector (Rheodyne, Rohnert Park, CA, USA) with an injection volume of 200 µL. After each injection, the surface was regenerated with an appropriate amount of HCl. The regeneration took 8–10 min after each injection. The end point of the measurement was considered as the data between 8 and 10 min, and the average of these data was used for the evaluation.

### 2.3. Functionalisation of the Sensor Surface

For the measurements, OW2400 glass type sensors were used. The OWLS measurement was carried out both in competitive and direct immunosensor formats. Since protein cannot be immobilised directly to the hydroxyl groups on the glass-type sensors, the sensor surface was modified using silanisation, using 10% of γ-aminopropyltriethoxysilane (APTS) in a liquid phase according to the method of Trummer et al. [35], to provide amino groups on the surface for covalent coupling. The immunoreagents were immobilised to the amino-modified sensor surface in a flow-through system. First, the surface was activated by injecting 200 µL of 2.5% glutaraldehyde in distilled water into the system, then the water was changed to 42 mM Tris (tris(hydroxymethyl)aminomethane) buffer (pH 7.4), with the subsequent injection of 10 µg/mL of GLY-HSA for the competitive assay format and 2.5 µg/mL anti-GLY antibody for the direct format. Washing off the excess and unbound molecules and also the sensor regeneration after each sample was performed by injecting 200 µL of 50 mM aqueous hydrochloric acid (HCl) when GLY-HSA was immobilised, but 10 mM aqueous hydrochloric acid was applied in case of direct measurement when anti-GLY antibodies were bound to the surface. After sensitisation, the sensor was ready for measurements.

The regeneration possibility and stability of the immunosensing surface are critical factors that can limit the reliability of the technique. Various procedures have been reported in the literature to mitigate non-specific adsorption through chemical modifications of the sensor surface [36,37]. In our previous research, we conducted experiments involving small-molecule proteins. Based on our experience, it is advisable to perform pre-running cycles with the biomolecules using immobilisation with glutaraldehyde. These protein-type molecules occupy any remaining free binding sites and also undergo homopolymerisation, resulting in a sufficiently stable attachment layer for the biomolecules.

Our experiments demonstrated that sensor signals measured for anti-GLY antibody detection exhibited sufficient and stable sensitivity after the pre-running cycles. As a result, approximately 15–18 samples could be consecutively measured on a single chip.

### 2.4. Derivatisation of the Glyphosate Standard

Since antibody formation is only initiated for immunogens larger than 5 kDa, glyphosate was linked to the carrier protein after N-modification for immunisation. Accordingly, the resulting antibody also shows the maximum affinity for derivatised glyphosate. In order to increase the sensitivity, before measurement the derivatisation of the standards and samples with an anhydrous acylating cocktail (90% acetic anhydride in acetonitrile) was required. In Eppendorf test tubes, 100 µL of standard/sample and 40 µL of the derivatisation mixture were gently homogenised and 100 µL of a 0.5 M aqueous sodium borate solution was added. The mixture was gently homogenised again and was incubated at room temperature for 20 min before it was ready for measurement. The samples were diluted by 42 mM Tris buffer (pH 7.4).

### 2.5. Fluorescent ELISA

The immunoassay protocol using the glyphosate ELISA kit (EK-102M200506) of the Institute of Isotopes Co., Ltd. (Budapest, Hungary) and the derivatised standards or samples was carried out according to the manufacturer’s protocol [28]. Briefly, 100 µL of the anti-GLY antibody (chicken polyclonal anti-glyphosate as the primary antibody) at a 0.4 µg/mL concentration was pipetted to 20 µL of the derivatised standards or samples in the wells of 96-well Costar HB microplates coated with 0.5 µg/mL glyphosate-analogue conjugate. The microplates were incubated for 2 h at room temperature and the wells were washed 4 times with 250 µL/well borate buffer. Then, 100 µL/well of goat anti-chicken IgY antibodies conjugated to horseradish peroxidase as a reporter enzyme (secondary antibody tracer) at 0.5 µg/mL concentration was added and plates were incubated for 30 min at room temperature, and the wells were washed 5 times with borate washing buffer. For fluorescent detection, in the final step, 100 µL/well of a solution (1:50:50 *v*/*v* mixture of ADHP, enhancer and peroxide) of QuantaRed Enhanced Chemifluorescent HRP Substrate Kit were added (Thermo Fisher Scientific Inc., Waltham, MA, USA), the plates were incubated for 5 min at room temperature, the enzymatic activity was stopped with 10 µL of QuantaRed Stop Solution and absorbances were read at a 531/593 nm wavelength using a SpectraMax iD3 Multi-Mode Microplate Reader (Molecular Devices, San Jose, CA, USA).

## 3. Results and Discussion

### 3.1. Direct Measurement of Glyphosate

An OWLS sensor was prepared by immobilising glyphosate-specific antibodies on the aminosilanised sensor surface for the direct measurement of glyphosate and signal response induced by the binding of the derivatised glyphosate standard to the immobilised antibody was measured. As for the immunisation, glyphosate conjugated to a carrier protein through the nitrogen atom of its glycine moiety was used. The resulting glyphosate-specific antibodies showed a high affinity to the N-modified hapten of glyphosate, therefore, for the measurement derivatised glyphosate standards were used.

For the measurement, the sensor surface was sensitised with 2.5 µg/mL glyphosate-specific antibody, and derivatised glyphosate standards were investigated in the concentration range of 0.001–100 ng/mL. The measurement was carried out under temperature control at 22 °C and a flow rate of 160 µL/min was used. The derivatised standards were diluted with 42 mM Tris buffer (pH set to 7.4 with hydrochloric acid) and were investigated at 100-fold final dilutions in order to eliminate the interfering effect of the derivatising agent on the measurement.

**Figure 1:**
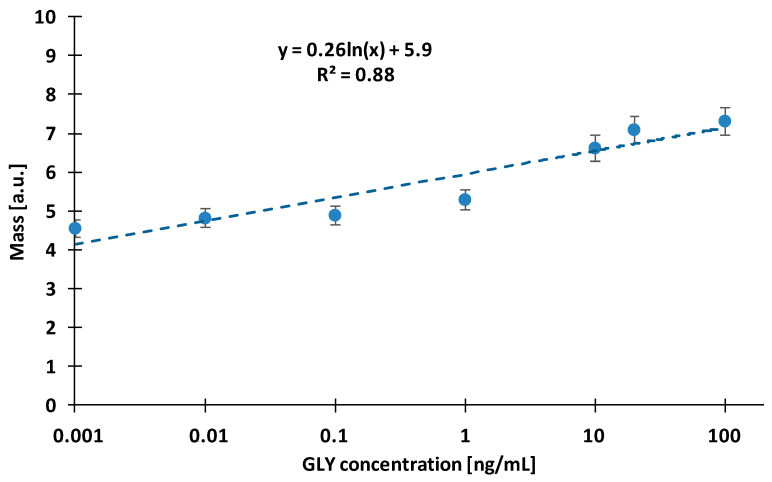
Standard calibration curve in the direct method with glyphosate-specific antibody immobilised on the sensor surface at 2.5 µg/mL concentration.

It can be seen in Figure 1 that by increasing the concentration of the glyphosate standard, the magnitude of the response signal did not increase significantly. The signals were small and unstable, causing a high relative deviation of the results. The surface of the sensor was rapidly saturated; therefore, it was concluded that the direct measurement method is not sufficiently sensitive for the determination of glyphosate.

### 3.2. Competitive Immunosensor

#### 3.2.1. Determination of the Optimal Immobilised Antigen Conjugate Concentration

To study the effect of the concentration of the coating antigen, glyphosate conjugated to human serum albumin (GLY-HSA) was immobilised, the surfaces of the sensor were treated with the conjugate at different concentrations (5, 10, 20 µg/mL). During measurement, a flow rate of 160 μL/min was applied and the sensor holder was temperature controlled at 22 °C. The glyphosate-specific antibody solution of 2.5 µg/mL (diluted in 42 mM Tris buffer, pH 7.4) was mixed at a 1:1 ratio with different concentrations of the derivatised and diluted standard solution (0, 10 ng/mL) and was injected into the sensor system.

**Figure 2:**
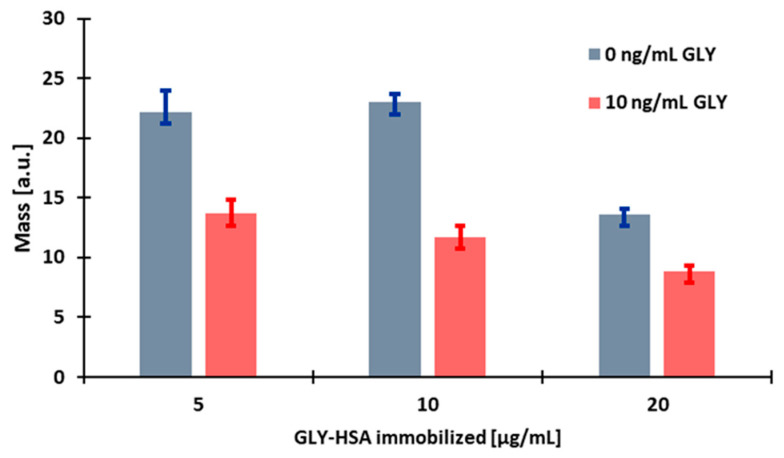
The effect of the concentration of glyphosate conjugated to human serum albumin (GLY-HSA) conjugate immobilised on the sensor surface in different concentrations (5, 10, 20 µg/mL) on the sensor response.

Comparing the responses of several sensors sensitised with different concentrations of GLY-HSA conjugate to antibody and standard, we found that when the conjugate was used at a concentration of 5 µg/mL, the magnitude of the response signal was adequate, but the stability of the sensor was poor, as shown by the large standard deviation in Figure 2. By increasing the amount of the conjugate to a concentration of 10 µg/mL, the response signals increased slightly in the case of the sample without glyphosate, and in the case of the sample containing glyphosate, the effect of inhibition of binding of the glyphosate-specific antibody was much more effective and the stability of the sensor also increased, e.g., for 5 µg/mL and for 10 µg/mL of the GLY-conjugate, the signal of the control antibody sample was 22.21 ± 1.76 a.u. and 23.00 ± 0.73 a.u., respectively. By further increasing the amount of the conjugate, the surface became overloaded and the responses for the glyphosate standard solution decreased significantly. As the immobilised layer will be thicker, spatial inhibition can be obtained; however, since in the present measurement technique the intensity of the signal decreases logarithmically with the distance from the sensor surface, the signal diminishes, e.g., for 20 µg/mL of the GLY-HSA conjugate the signal obtained with control antibody decreased to 13.62 ± 0.49 a.u. Based on the results, 10 µg/mL GLY-HSA conjugate was immobilised in further experiments.

#### 3.2.2. Determination of the Amount of the Applied Antibody

**Figure 3:**
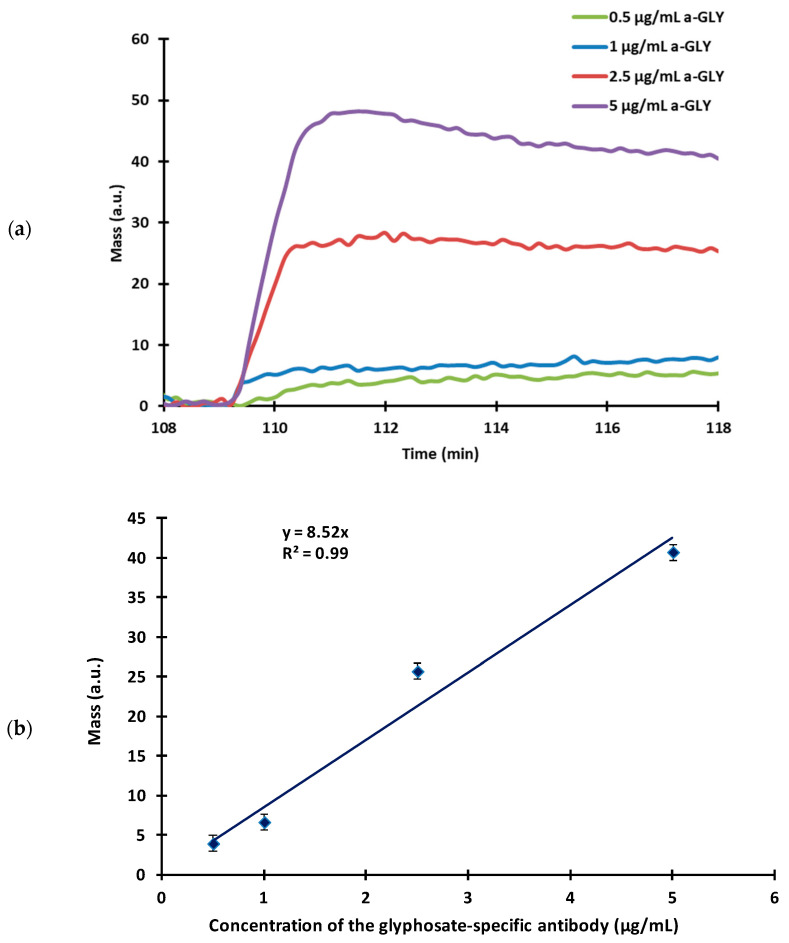
(**a**) The sensor responses of the glyphosate-specific antibody (a-GLY) at different concentrations (5 µg/mL, 2.5 µg/mL, 1 µg/mL, 0.5 µg/mL) using a sensor surface modified with 10 µg/mL GLY-HSA conjugate. (**b**) A titration curve of the sensor signal as a function of the antibody concentration.

The amount of antibody used is one of the most influencing factors that determines the sensitivity of the sensor. For the measurement, the sensor surface was sensitised with 10 µg/mL of GLY-HSA conjugate at optimal conditions (a flow rate of 160 µL/min, room temperature) and the glyphosate-specific antibodies were injected onto the sensor surface at increasing concentrations (0.5–5 µg/mL) to assess their binding affinity. For the optimal operation of the immunosensor, antibody concentrations that just saturate the surface of the sensor must be chosen, which coincides with the lowest concentration still giving a distinguishable signal. The typical signal kinetic curves and a titration curve are presented in Figure 3a,b, respectively.

The use of low antibody concentrations (0.5–1 µg/mL) slightly saturated the sensor surface, resulting in nearly identical, small, uncertain signals. The sensitivity of the sensor was remarkable in the case of standard measurement, but unfortunately only at a cost of a reduced signal. By further increasing the antibody concentration, more stable signals were obtained that were clearly distinguishable and of adequate magnitude. At a concentration of 5 µg/mL after the signal reached its highest peak intensity, a washout could be observed, meaning that too much antibody was present in the system, which was not able to bind to the antigen-sensitised surface. At high antibody concentrations, sufficiently large signals were obtained, but at the same time the sensitivity of the method decreased. Determination of the optimal concentration of the working antibody is a sensitive equilibrium, therefore, considering the height and shape of the signals, the optimal working concentration was chosen to be 2.5 µg/mL.

For the competitive measurement, GLY-HSA conjugate at a concentration of 10 µg/mL was immobilised with 2.5% glutaraldehyde solution on the surface. Derivatised and diluted standards were mixed with antibodies at a concentration of 2.5 µg/mL in a 1:1 ratio, and the mixtures were subsequently injected into the system. Measurements were carried out at room temperature with a flow rate of 160 µL/min applied.

The excess of the derivatising agent after the derivatisation step could react with the immobilised antibody or antigen–conjugate, therefore, we consistently utilized a diluted step after derivatisation. Moreover, the course of the measurements, the curve exhibited a considerable increase following the injection of the 10× diluted derivatising agent due to refractive index alteration. Nonetheless, after the subsequent washing step, this signal increased due to the refractive index change being diluted out. Such an initial increase was negligible after the injection of the 100× diluted derivatising agent. Therefore, a dilution of 1:100 was deemed necessary for signal stability.

**Figure 4:**
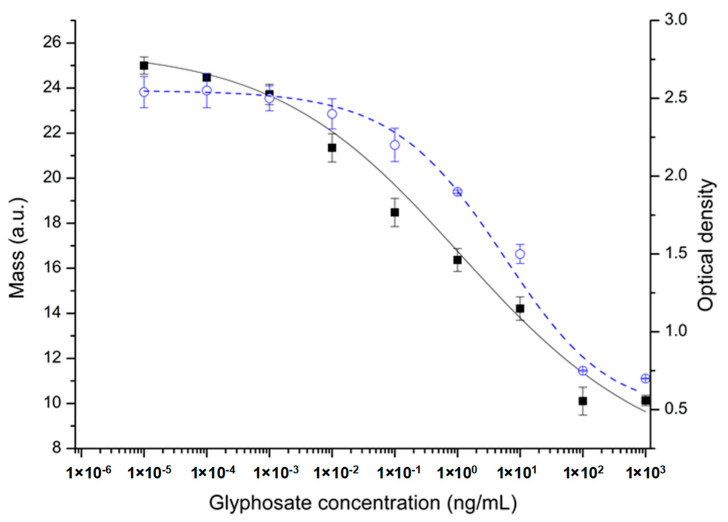
Standard calibration curves of the OWLS immunosensor (10 µg/mL GLY-HSA, 2.5 µg/mL antibody, signal in mass in arbitrary units, statistical parameters: r^2^ = 0.99, EC_50_ = 0.604 ng/mL) (black rectangles, black solid line) and the ELISA determination (using the ELISA kit according to manufacturer’s protocol signal in optical density at 405 nm wavelength, statistical parameters: r^2^ = 0.98, EC_50_ = 10.7 ng/mL) (blue open circles, blue dashed line).

Analytical standard curves were obtained using a concentration series of glyphosate standards in a range of 10^−4^–10^3^ ng/mL, where its inhibitory effect on antibody binding to the conjugate of the antigen immobilised on the waveguide surface via covalent coupling was detected and was compared to that obtained by ELISA (Figure 4). The dynamic detection range of glyphosate was found in the 0.001–100 ng/mL glyphosate concentrations and an LOD of 0.0001 ng/mL for glyphosate was obtained. This range is two orders of magnitude lower than that obtained in the direct OWLS method. Detection sensitivity showed a low EC_50_ value of 0.604 ng/mL, corresponding to an improvement in the detection range compared to the ELISA method that applied visual absorbance signal detection, where the EC_50_ value of 10.7 ng/mL glyphosate was obtained [28].

Among the sample standards, we consistently measured a blank sample and compared the signal of the standards to it. Although the signal magnitudes slightly decreased during the measurements, the difference between the standards and the blank sample remained stable. Specifically, the difference of the standard sample with a concentration of 0.1 ng/mL and the blank sample was determined to be 9.97 ± 0.82 mass/area unit based on 9 parallel measurements.

### 3.3. Cross-Reactivity of the Glyphosate-Specific Antibody

GLY is a secondary amine due to its central nitrogen atom that contains a hydrogen atom and two alkyl-type substituents (a phosphonomethyl group and a carboxymethyl group). In contrast, in addition to the two alkyl groups, the haptenic derivative of GLY contains a succinyl group for further conjugation on the central nitrogen atom and does not possess an N–H bond. The results of the study indicated that the antibodies raised against the immunogen conjugate of the haptenic derivative of GLY exhibited distinctive specificity towards this chemical difference between GLY and its hapten. Specifically, these antibodies did not bind to the secondary amine GLY but demonstrated strong binding to its acetylated derivative, acetyl-GLY. Interestingly, the same antibodies did not display observable cross-reactivity to a closely related commercial derivative, PMIDA, which is a tertiary amine with three alkyl-type substituents (a phosphonomethyl group and two carboxymethyl groups) on its central nitrogen atom. The specificity of the glyphosate-specific antibody produced in chickens was compared with compounds structurally related to glyphosate by examining the binding affinity. Derivatisation was also performed on the individual samples. Compounds tested for cross-reactivity (CR) included the major glyphosate metabolite N-aminomethylphosphonic acid (AMPA), the (N-(phosphonolmethyl)iminodieacetic acid (PMIDA), glycine and acetylglycine (Table 1). Based on the results, we proved that compounds structurally similar to glyphosate can only affect the measurements at extremely high concentrations and the antibody is sufficiently selective for glyphosate.

### 3.4. Determination of GLY Concentration in Surface Water Samples

The optimised competitive immunosensor was applied to determine GLY concentrations in different surface water samples, aiming to assess the presence of a matrix effect. Surface water samples were collected from lakes and a river in Hungary. Prior to their application in the novel OWLS immunosensor, the GLY residues of the surface water samples were determined by the fluorescent ELISA method [28]. For the competitive fluorescent ELISA method, the GLY concentration was measured from undiluted surface water samples, while a 1:100 dilution was used for the derivatised samples, when using the OWLS technique. Based on our results, the GLY residue concentration was found below the detection limit in the three surface waters that were examined. In order to investigate the matrix effect, the water samples were spiked with a known amount of GLY, and the concentration was measured. As shown in Table 2, we can conclude that there was no matrix effect observed in the samples. The measured concentrations using both analytical systems corresponded well to the spiked concentration.

## 4. Conclusions

Novel label-free optical waveguide light-mode spectroscopy (OWLS) immonosensor setups were devised in direct and competitive formats for the detection of the herbicide active ingredient glyphosate, using polyclonal antibodies used in a flow-injection analyser system (at a flow rate of 160 µL/min). As the antibodies were generated against a hapten derivatised (acylated) on the nitrogen atom of glyphosate, both sensor formats relied on derivatisation (acetylation) of the analyte prior to immunosensing. While the direct immunosensor prepared by immobilising the antibodies at a 2.5 µg/mL concentration on an aminosilanised sensor surface was found not sufficiently sensitive for the determination of glyphosate at or below 100 ng/mL under the assay conditions studied, the immunosensor in the competitive format allowed remarkable sensitivity for glyphosate. After immobilising a human serum albumin conjugate of glyphosate on the sensor surface using a conjugate solution of 10 µg/mL concentration and applying the polyclonal antibody at 2.5 µg/mL, glyphosate could be quantitatively determined in the competitive immunosensor format in the detection range of 0.01–100 ng/mL and an EC_50_ value of 0.60 ng/mL. Comparing the different measurement methods (Table 3), it can be concluded that the linear measuring range and LOD of the newly developed OWLS based immunosensor correspond to the measing parameters available with similar methods and are sufficiently sensitive for the examination of different samples. No significant cross-reactivity was observed with structurally related glyphosate metabolites or derivatives, and the immunosensor allowed better detection sensitivity than the corresponding ELISA method with visual absorbance signal detection using the same antibody.

## Figures and Tables

**Table 1:** Percentage of cross-reactivity of the competitive OWLS immunosensor and the corresponding ELISA method with glyphosate and its derivatives.

Compound	Nominal Conc. (ng/mL)	Detected Conc. OWLS (ng/mL)	Detected Conc. OWLS (%)	Detected Conc. Fluorescent ELISA (ng/mL) [28]	Detected Conc. Fluorescent ELISA (%)
Glyphosate *	100	97.6 ± 1.3	100	99.3 ± 0.8	100
	50	51.7 ± 2.1	100	50.4 ± 1.1	100
AMPA *	6700	<0.1	<0.1	<0.01	<0.0015
	100	<0.1	<0.1	<0.01	<0.01
PMIDA *	1650	0.22	0.2	0.31	0.018
Glycine	100	<0.1	0.15	<0.1	<0.01
Acetylglycine	100	<0.1	0.15	<0.1	<0.01

* Chemical names: glyphosate—N-(phosphonomethyl)glycine; AMPA—N-aminomethylphosphonic acid; PMIDA—N-(phosphonolmethyl)iminodiacetic acid.

**Table 2:** Comparison of GLY concentration of surface water samples measured by OWLS and fluorescent ELISA.

Sample ID	Spiked Conc. (ng/mL)	Conc. OWLS (ng/mL)	Recovery OWLS (%)	Concentration Fluorescent ELISA (ng/mL) [28]	Recovery Fluorescent ELISA (%)
1	0	<0.01		<0.09	
2	0	<0.01		<0.09	
3	0	<0.01		<0.09	
4	0.1	0.080 ± 0.009	80.0 ± 9.0	0.112 ± 0.012	112.0 ± 12.0
5	1.56	1.67 ± 0.24	107.1 ± 15.4	1.46 ± 0.22	93.6 ± 14.1
6	12.5	11.8 ± 0.9	94.4 ± 7.2	14.1 ± 1.04	112.8 ± 8.3
7	100	106.3 ± 7.6	106,3 ± 7.6	108.1 ± 6.3	108.1 ± 6.3

**Table 3:** Comparative analysis among glyphosate detection methods.

Method	Linear Measuring Range	LOD	Matrix	Reference
HPLC-UV	0.3–48.5 µg/mL	0.009 µg/mL	Water	[22]
LC-MS/MS	0–50 ng/mL	0.5 ng/mL	Breast milk	[23]
LC-ESI-MS/MS	50–500 pg/mL 0.05–0.5 mg/kg	5 pg/mL 5 µg/kg	Water Soil	[24]
LC-ESI-MS/MS	10–400 µg/kg or 10–800 µg/kg	0.09–0.8 µg/kg	Apple Mushrooms Grapefruit Linseed Red lentils Wheat	[25]
OWLS	0.01–100 ng/mL	0.0001 ng/mL	Water	Present study
Fluorescent ELISA	0–100 ng/mL	0.09 ng/mL	Water	[28]
FLIS *	0–1 ng/mL	0.021 ng/mL	Water	[38]

* FLIS—Fluorescent immunosensor.

## Data Availability

The data presented in this study are available on request from the corresponding author. The data are not publicly available due to privacy reasons.
